# Supplementary material for: A UMLS-based spell checker for natural language processing in vaccine safety
Source: BMC Med Inform Decis Mak. 2007 Feb 12;7:3. doi: 10.1186/1472-6947-7-3 (PMC1805499; doi:10.1186/1472-6947-7-3)
Supplement: Additional file 13 — RAP application directory. Sets up directory for RAP (RDF application for PHP) source code [file 1472-6947-7-3-S13.gz › rap/doc/phpdoc/model/Resource.html]

xml version="1.0" encoding="iso-8859-1"?


Docs For Class Resource


## Class Resource

Description

Description |
Descendents
| Methods (details)

An RDF resource.

Every RDF resource must have a URIref. URIrefs are treated as logical constants, i.e. as names which denote something (the things are called 'resources', but no assumptions are made about the nature of resources.) Many RDF resources are pieces of vocabulary. They typically have a namespace and a local name. In this case, a URI is composed as a concatenation of the namespace and the local name.

History:

- 11-09-2003 : Fixed bug in equals() <radol@gmx.de>
- 09-30-2002 : Fixed bug in getNamespace() and getLocalName().
- 09-10-2002 : First version of this class.

- access: public
- todo: nothing
- author: Chris Bizer <chris@bizer.de>
- version: V0.9.1

Located in /model/Resource.php (line 30)

```
Object
   |
   --Node
      |
      --Resource
```

Direct descendents

Description |
Descendents
| Methods (details)

| Class | Description |
| --- | --- |
| BlankNode | An RDF blank node. |
| ResResource | An RDF Resource. |

Method Summary

Description |
Descendents |
Methods (details)

Resource
Resource
(string $namespace\_or\_uri, [string $localName = NULL])

boolean
equals
(object resource $that)

string
getLabel
()

string
getLocalName
()

string
getNamespace
()

string
getURI
()

string
toString
()

Methods

Description |
Descendents |
Methods (details)

Constructor Resource (line 48)

Constructor Takes an URI or a namespace/localname combination

- access: public

Resource

Resource
(string $namespace\_or\_uri, [string $localName = NULL])

- string
  $namespace\_or\_uri
- string
  $localName

equals (line 116)

Checks if the resource equals another resource.

Two resources are equal, if they have the same URI

- access: public

boolean

equals
(object resource $that)

- object resource
  $that

---

Redefined in descendants as:

- BlankNode::equals()
  : Checks if two blank nodes are equal.
- ResResource::equals()
  : Checks if the resource equals another resource.

getLabel (line 71)

Returns the label of the resource, which is the URI of the resource.

- access: public

string

getLabel
()

---

Redefined in descendants as:

- BlankNode::getLabel()
  : Returns the ID of the blank node.

getLocalName (line 92)

Returns the local name of the resource.

- access: public

string

getLocalName
()

getNamespace (line 80)

Returns the namespace of the resource. May return null.

- access: public

string

getNamespace
()

getURI (line 62)

Returns the URI of the resource.

- access: public

string

getURI
()

toString (line 104)

Dumps resource.

- access: public

string

toString
()

---

Redefinition of:

Object::toString()
:   Serializes a object into a string

---

Redefined in descendants as:

- BlankNode::toString()
  : Dumps bNode.

#### Inherited Methods


Inherited From Object

> Object::toString()

Documentation generated on Thu, 7 Jul 2005 13:43:45 +0200 by phpDocumentor 1.3.0RC3
